# Determination of an RT-qPCR viral load cutoff point for the etiologic diagnosis of rotavirus A diarrhea in neonate dairy calves

**DOI:** 10.3389/fvets.2022.952197

**Published:** 2022-08-12

**Authors:** Rubén D. Caffarena, Matías Castells, Carlos O. Schild, María L. Casaux, Joaquín I. Armendano, Rodney Colina, Federico Giannitti

**Affiliations:** ^1^Plataforma de Investigación en Salud Animal, Instituto Nacional de Investigación Agropecuaria (INIA), Estación Experimental La Estanzuela, Colonia, Uruguay; ^2^Unidad Académica Salud de los Rumiantes, Departamento de Producción Animal, Facultad de Veterinaria, Universidad de la República, Montevideo, Uruguay; ^3^Laboratorio de Virología Molecular, Departamento de Ciencias Biológicas, Centro Universitario Regional (CENUR) Litoral Norte, Universidad de la República, Salto, Uruguay; ^4^Facultad de Ciencias Veterinarias, Universidad Nacional del Centro de la Provincia de Buenos Aires (UNCPBA), Tandil, Argentina

**Keywords:** clinical outcome, dairy calves, enzyme-linked immunosorbent assay (ELISA), rotavirus A, neonatal calf diarrhea, RT-qPCR—real-time quantitative polymerase chain reaction, viral load, cutoff point

## Abstract

Rotavirus A (RVA) is amongst the most widespread causes of neonatal calf diarrhea. Because subclinical infections are common, the diagnosis of RVA-induced diarrhea cannot rely solely on molecular viral detection. However, RT-qPCR allows for quantification of RVA shedding in feces, which can be correlated with clinical disease. Here, we determine an optimal cutoff of rotaviral load quantified by RT-qPCR to predict RVA causality in diarrheic neonate calves, using RVA antigen-capture ELISA as reference test. Feces from 328 diarrheic (*n* = 175) and non-diarrheic (*n* = 153), <30-day-old dairy calves that had been tested by ELISA and tested positive by RT-qPCR were included. Of 82/328 (25.0%) ELISA-positive calves, 53/175 (30.3%) were diarrheic, whereas 124/153 (81.0%) non-diarrheic calves tested negative by ELISA. The median log10 viral load was significantly higher in diarrheic vs. non-diarrheic and ELISA-positive vs. -negative calves, indicating a higher viral load in diarrheic and ELISA-positive calves. A receiver operating characteristic (ROC) analysis was conducted using the viral loads of the 175 diarrheic calves that had tested either positive (*n* = 53, cases) or negative (*n* = 122, controls) by ELISA. The optimal log10 viral load cutoff that predicted RVA causality in diarrheic calves was 9.171. A bootstrapping procedure was performed to assess the out-of-bag performance of this cutoff point, resulting in sensitivity = 0.812, specificity = 0.886, area under the curve = 0.922, and positive and negative diagnostic likelihood ratios of 11.184 and 0.142, respectively. The diagnostic accuracy of the cutoff was excellent to outstanding. This information will help in the interpretation of RVA RT-qPCR results in feces of diarrheic calves submitted for laboratory testing.

## Introduction

Neonatal calf diarrhea is the leading cause of death in dairy calves (1, 2) and has long-term productive repercussions in survivors, such as stunting, increased age at first calving and reduced milk production in the first lactation (3), resulting in significant economic losses to the livestock industry (4). Although neonatal calf diarrhea can be multifactorial, rotavirus A (RVA, *Reoviridae*) is amongst the most frequent and widespread causative enteropathogens.

After fecal-oral transmission, rotaviruses establish infection and replicate in the mature villus enterocytes of the proximal small intestine. Lysis of the infected enterocytes causes release of large numbers of virions into the intestinal lumen, leading to further infection and damage of villus enterocytes in distal segments of the small intestine. As viral multiplication progresses, damaged villus enterocytes slough off and are replaced by immature enterocytes migrating from the crypts. This alters the ratio of intestinal absorption and secretion, leading to accumulation of fluid (and virions) in the intestinal lumen with subsequent diarrhea (5). Thus, individuals suffering from diarrhea caused by RVA are expected to shed significantly higher viral loads than asymptomatic shedders (6). Similarly, calves with diarrhea caused by RVA are also expected to shed significantly higher rotaviral loads than those with diarrhea caused by other etiologies.

The etiologic diagnosis of rotaviral diarrhea depends upon viral identification in feces. However, as asymptomatic or subclinical individuals can shed rotavirus, viral identification alone does not warrant disease causality and interpretation of laboratory test results in clinical settings is not always straightforward. A variety of diagnostic techniques have been used in humans and animals to detect rotavirus particles, antigens, or RNA in feces, including electron microscopy, enzyme-linked immunosorbent assay (ELISA), immunofluorescence assays, lateral flow immunochromatography (LFIC), reverse transcription conventional PCR (RT-PCR) or reverse transcription real-time quantitative PCR (RT-qPCR) (7, 8).

RT-qPCR assays have been increasingly applied in the diagnosis of viral diseases in veterinary diagnostic laboratories as they have a fast turnaround time, high analytical sensitivity, reproducibility, and reduced risk of cross-contamination compared to other molecular techniques (i.e., conventional PCR). Unlike other diagnostic tests such as ELISA, LFIC and conventional PCR, RT-qPCR assays yield a continuous rather than a binary (positive or negative) outcome, the cycle of quantification (Cq) value, which may be used not only to determine if a target sequence is present in a sample but also for its quantification (9). The Cq value is inversely proportional to the logarithmic concentration of the target nucleic acid originally present in a sample; therefore, the lower the Cq value the higher the viral load and vice versa (9, 10). Because the relationship between the Cq value and the viral load is not linear, the Cq value should not be used as a direct measure of the viral load; however, using standard quantification curves, the Cq value can be used to calculate the viral load (viral genome copies per ml or gram of sample), rendering results that are more comparable between samples (11).

Generally, when a Cq value is obtained, the sample is considered positive; however, given the high sensitivity of RT-qPCR assays and the considerable percentage of clinically healthy or subclinical individuals that shed rotavirus in feces (12–14), caution should be taken when interpreting positive (qualitative) RT-qPCR results in clinical settings (10). In this context, using RT-qPCR to quantify the viral load represents an advantage with direct clinical application. However, applying RT-qPCR as a routine diagnostic tool for the investigation of diarrhea caused by rotavirus requires establishing the optimal viral load cutoff value to distinguish symptomatic from asymptomatic shedders, to distinguish calves with diarrhea caused by rotavirus from those with diarrhea caused by etiologies different from rotavirus (9, 10, 15), or false positive results with high Cq values caused by degradation of the probe-based fluorophore due to cross contamination or by nonspecific amplification of nucleic acids (9).

Considering that RVA positivity by ELISA has been associated with diarrhea in dairy calves (16) and that RVA viral load and ELISA are highly correlated with diarrheic disease in humans (10), the aim of this work was to define an optimal RVA RT-qPCR viral load cutoff point to establish RVA as the sufficient cause of diarrhea in neonate dairy calves, using a commercial antigen-capture ELISA as a reference test. If the results of the ELISA can be predicted accurately by using a RVA RT-qPCR viral load cutoff point, then these tests could be used interchangeably. The information generated in this study will help in the diagnostic investigation and interpretation of RVA RT-qPCR results in fecal samples of diarrheic calves submitted for laboratory testing.

## Materials and methods

### Sample selection and inclusion criteria

Fecal samples from two previous studies by our group (6, 16) were eligible for this study. Samples were included if (a) they belonged to <30-day-old dairy calves with known clinical status (diarrheic or non-diarrheic), (b) had been tested by a commercial antigen-capture ELISA for the detection of RVA regardless of the result (see below), and (c) had yielded a positive (Cq) result for RVA by RT-qPCR (see below). A total of 328 samples from 175 diarrheic and 153 non-diarrheic calves met the inclusion criteria.

### Antigen-capture ELISA for the detection of RVA

We used the commercial kit Pathasure Enteritis 4 (Biovet Inc., St-Hyacinthe, Canada) according to the manufacturer's specifications (16). Briefly, 0.5 ml of each fresh stool sample was diluted in 4.5 ml of 1× washing solution (1:10 dilution). Two drops (~100 μl) of the resulting solution per sample, and ready-to-use positive and negative controls, were added to individual wells of the ELISA plate coated with anti-rotavirus monoclonal antibody, and incubated at 23 ± 2°C for 30 min. After incubation, the wells were emptied and washed 5 times with the same 1× washing solution, before drying the plate by tapping it on absorbent paper. Two drops (~100 μl) of ready-to-use anti-rotavirus conjugate was added to each well and incubated for 30 min. at 23 ± 2°C. After washing and drying as described, three drops (~150 μl) of substrate were added to each well. After an incubation of 10 min. at 23 ± 2°C away from light, the results were read with the naked eye. A positive result was obtained if the intensity of color in a well was similar to the one obtained in the well with positive control, otherwise the sample was considered negative.

### RT-qPCR assay for the detection and quantification of RVA

This test was performed as previously described (6) on an aliquot of each fecal sample stored in a freezer at −20°C. Briefly, after thawing, feces were diluted 1:10 (v:v) in phosphate buffered saline solution and centrifuged at 3,000 g for 20 min. at 4°C; supernatants were stored at −80°C. RNA was extracted using the QIAamp^®^ Cador^®^ Pathogen Mini Kit (Qiagen^®^), following the manufacturer's instructions with an elution volume of 50 μl. RevertAid Reverse Transcriptase (Thermo Fisher Scientific^®^) and random hexamer primers (Qiagen^®^) were used for reverse transcription. qPCR was performed with TaqMan^®^ technology and Rotor-Gene Q instrument (Qiagen^®^). Five μl of cDNA as template, SensiFAST™ Probe No- ROX Kit (Bioline^®^), primers and probe with a final concentration of 0.4 and 0.2 μmol/L^−1^, respectively, and DNase/RNase free water to a final volume of 25 μl, were used. RVA-positive and -negative fecal samples were used as positive and negative controls to validate the results. Samples and controls were analyzed in duplicates, and the average Cq value obtained for both duplicates was used to calculate the viral load (genome copies per ml of feces) using a standard curve exactly as described by Castells et al. (17). A log10 transformation of this value was used for the results and statistical analyses.

### Statistical analysis

The result of the ELISA (positive or negative), and log10 viral load for each calf were entered into a Microsoft Excel 2013 spreadsheet, and imported into SAS Studio (SAS Institute Inc., Cary, NC) for statistical analyses. Due to the lack of normal distribution, the log10 viral load was expressed as the median with the interquartile range (IQR) for diarrheic and non-diarrheic ELISA-positive and -negative calves. The Wilcoxon rank sum (non-parametric) test was used to assess whether there were differences in the median log10 viral load between diarrheic and non-diarrheic calves, and between calves that tested positive or negative by ELISA.

A receiver operating characteristic (ROC) analysis was performed with R software v3.6.2 (http://www.r-project.org/) to calculate the optimal log10 viral load cutoff point that would distinguish calves with diarrhea caused by RVA from calves with diarrhea not associated with RVA infection using the antigen-capture ELISA as reference test (8, 18, 19) through the maximization of the Youden index (diagnostic sensitivity + diagnostic specificity – 1) (9, 20). For the ROC analysis, all 175 diarrheic calves were included; ELISA-positive calves (*n* = 53) were defined as cases, whereas ELISA-negative calves (*n* = 122) were considered controls. Additionally, to estimate the performance that the cutoff point would have in a hypothetically different dataset than the one used to actually calculate the cutoff point (out-of-bag performance), a bootstrapping procedure was performed (21).

For the statistical analyses, a *p*-value < 0.05 was considered significant. Graphics were built with R software.

## Results

Of the 328 samples that met the inclusion criteria, 82 (25.0%) were ELISA-positive, of which 53 (64.6%) were from diarrheic and 29 (35.4%) from non-diarrheic calves. The remainder 246 (75.0%) samples were ELISA-negative, of these 122 (49.6%) were from diarrheic and 124 (50.4%) from non-diarrheic calves. The log10 viral load of the 328 samples ranged from 4.2 to 12.9. The median, minimum and maximum log10 viral load and the IQR in diarrheic and non-diarrheic calves, as well as in calves that tested positive or negative by ELISA are shown in Table 1. There were statistically significant differences in the median log10 viral load between calves with and without diarrhea, and between ELISA-positive and -negative calves (Table 1).

**Table 1:** Log10 viral load of RVA determined by RT-qPCR in calves with and without diarrhea, and in calves that tested positive or negative for RVA antigen by capture ELISA.

		***n***	**Median**	**Min**.	**Max**.	**IQR**	*p* value*
Diarrhea	No	153	6.3	4.3	12.9	3.6	<0.02
	Yes	175	7.6	4.2	12.6	4.6	
ELISA	Negative	246	6.1	4.2	12.1	2.4	<0.0001
	Positive	82	11.2	4.6	12.9	1.8	

IQR, interquartile range.

* Wilcoxon rank sum test.

**Figure 1:**
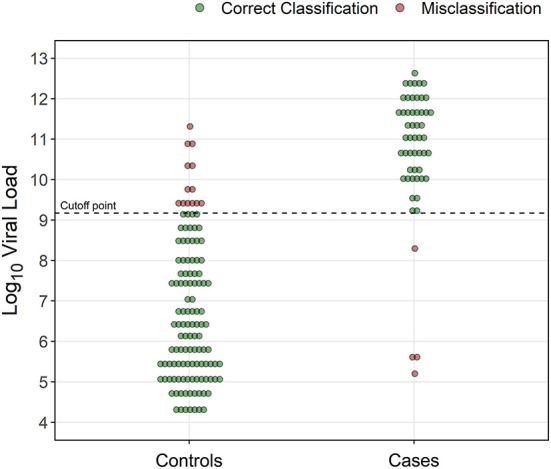
Rotavirus A log10 viral load determined by RT-qPCR in fecal samples of 175 diarrheic calves that tested negative (*n* = 122, controls) or positive (*n* = 53, cases) by ELISA, and discrimination ability of the cutoff point (dotted line). Each sample represents a calf sample.

**Figure 2:**
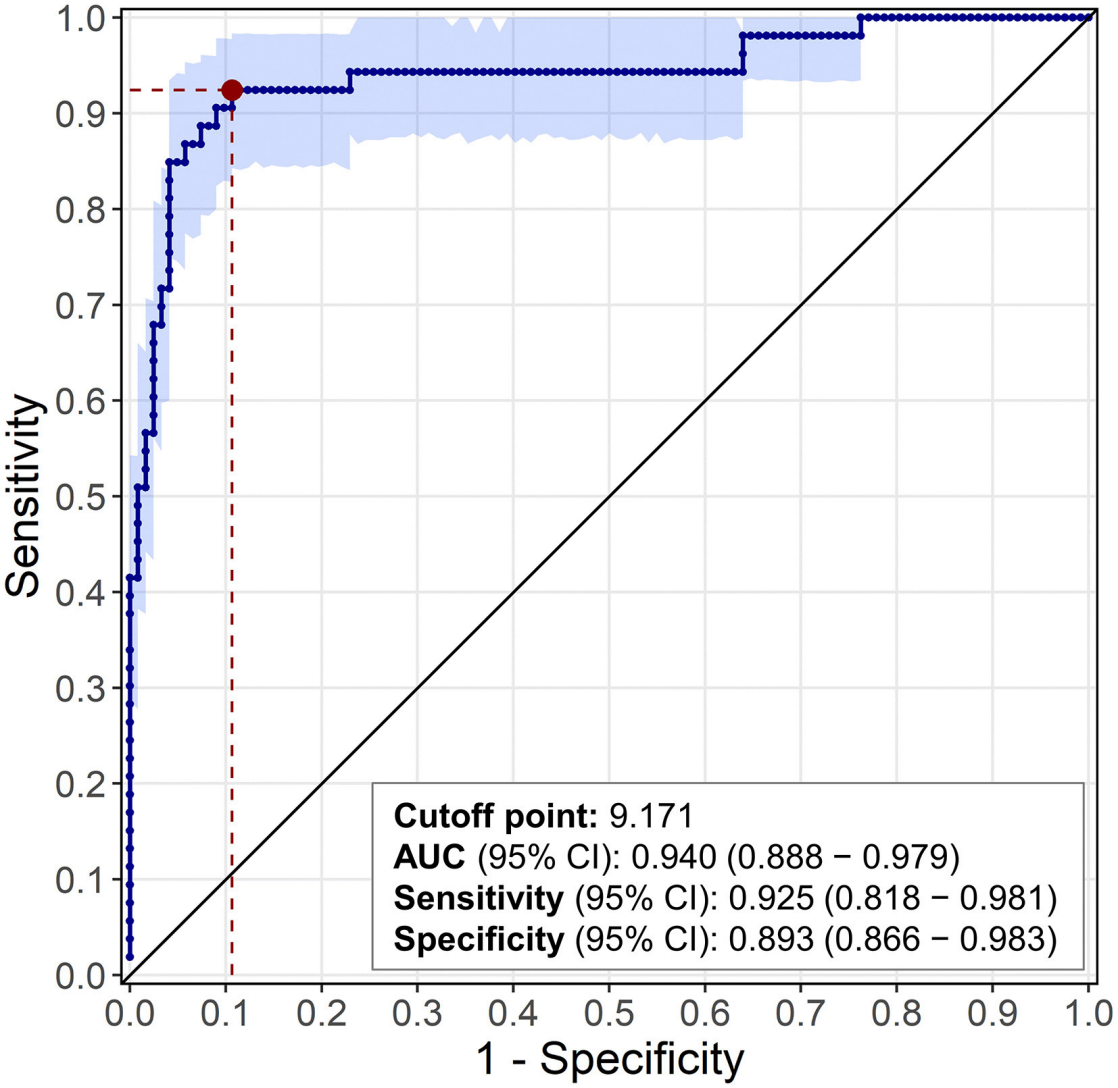
Graphical plot of the ROC curve. Sensitivity = true positive rate; 1 – specificity = false positive rate.

Of the 328 samples included in the study, 175 met the defined case/control criteria for the ROC analysis, including 53 ELISA-positive diarrheic cases and 122 ELISA-negative diarrheic controls. There was a statistically significant difference (*p* < 0.0001) in the median log10 viral load between cases (11.1, IQR: 1.6) and controls (6.3, IQR: 2.7), with very little overlapping in the distribution of viral load in these two groups, which had a clear bimodal pattern. The optimal cutoff point at which the log10 viral load was associated with diarrhea in ELISA-positive calves (cases) was 9.171 (Figure 1). The sensitivity, specificity, positive and negative predictive values, positive and negative diagnostic likelihood ratios, and area under the ROC curve (Figure 2) are shown in Table 2. As indicated by the positive diagnostic likelihood ratio (DLR+), cases (ELISA-positive diarrheic calves) were 8.676 times more likely to have log10 viral loads >9.171 than controls (ELISA-negative diarrheic calves). The outputs of the bootstrapping procedure to assess the out-of-bag performance of the cutoff point are shown in Table 3.

**Table 2:** Results of the receiver operating characteristic analysis for RVA RT-qPCR using antigen-capture ELISA as a reference test for RVA detection in feces of 175 diarrheic calves that tested negative (*n* = 122, controls) or positive (*n* = 53, cases) by ELISA.

**Youden Index**	**Cutoff point (log_10_ viral load)**	**Sensitivity (95% CI)**	**Specificity (95% CI)**	**DLR+ (95% CI)**	**DLR– (95% CI)**	**AUC (95% CI)**
0.812	9.171	0.925 (0.818–0.981)	0.893 (0.866–0.983)	8.676 (6.887–52.277)	0.084 (0.021–0.192)	0.940 (0.888–0.979)

CI, confidence interval (percentile bootstrap method); DLR, diagnostic likelihood ratio; AUC, area under the curve.

**Table 3:** Results of the bootstrapping procedure to assess the out-of-bag performance of the RVA RT-qPCR optimal cutoff point (log10 viral load of 9.171).

**Sensitivity_oob_ (95% CI)**	**Specificity_oob_ (95% CI)**	**DLR+_oob_ (95% CI)**	**DLR–_oob_ (95% CI)**	**AUC_oob_ (95% CI)**
0.812 (0.680–1.000)	0.886 (0.825–1.000)	11.184 (5.155–infinity)	0.142 (0.000–0.341)	0.922 (0.879–0.992)

CI, confidence interval (percentile bootstrap method); DLR, diagnostic likelihood ratio; AUC, area under the curve; oob, out-of-bag.

All values are expressed as median.

## Discussion

In this study we determined, for the first time, an optimal viral load cutoff point to establish an association between RVA shedding in feces and diarrhea caused by this pathogen in neonatal dairy calves. As determined by the AUC of the ROC analyses using the actual dataset as well as the bootstrapping procedure, the RT-qPCR test we used had an excellent to outstanding diagnostic accuracy, with >92% chance to discriminate ELISA-positive diarrheic calves from ELISA-negative diarrheic controls (22). In this context, in a diagnostic laboratory setting, the commercial ELISA test could be replaced by (or used interchangeably with) the RT-qPCR assay in the etiologic diagnosis of RVA in diarrheic calves.

Additionally, we performed a second ROC analysis (data not shown) using the viral loads of the same 53 ELISA-positive diarrheic calves (cases) and all other calves (*n* = 275) regardless of their clinical status and ELISA result (controls). Interestingly, this approach revealed the same optimal log10 viral load (9.171) as the cutoff point that would best discriminate ELISA-positive diarrheic calves from all other calves included in the study. Thus, this cutoff can be used to predict RVA-associated diarrhea in calves of unknown clinical status, although with a slightly lower accuracy as determined by the bootstrapping procedure to assess the out-of-bag performance [sensitivity = 0.842 (95% CI, 0.706–1.000), specificity = 0.807 (95% CI, 0.765–0.889), AUC = 0.856 (95% CI, 0.814–0.935), DLR+ = 5.128 (95% CI, 3.709–7.651), DLR– = 0.134 (95% CI, 0.000–0.346)] than in diarrheic calves. When the ROC analysis is conducted using the 153 non-diarrheic calves, 29 of which were positive by ELISA, the log10 viral load cutoff value that would best discriminate asymptomatic shedders from asymptomatic non-shedders as determined by ELISA is 10.034, with the following out-of-bag performance: sensitivity = 0.784 (95% CI, 0.571–1.000), specificity = 0.911 (95% CI, 0.757–1.000), AUC = 0.902 (95% CI, 0.856–0.996), DLR+ = 13.933 (95% CI, 3.833–infinity), DLR– = 0.146 (95% CI, 0.000–0.449).

The availability of ELISA-negative diarrheic (control) calves was a practical aspect of our work, which even with a relatively small sample size, allowed us to interpret the global results of the RT-qPCR and estimate an optimal cutoff to associate the results of a single molecular test with RVA causality in diarrheic calves (10).

Diagnostic tests based on the detection of antigens such as capture ELISA need a large amount of free antigen to generate a positive reaction, therefore, although less sensitive, a positive result is more likely to be clinically meaningful regarding causality (23–25). Likewise, the magnitude of viral shedding (viral load) can help to determine causality (10, 25), providing the clinician with additional information that can predict with more confidence whether the virus is responsible for the disease in clinical cases of diarrhea in field investigations (24).

The DLR is defined as the ratio between the probability of a positive result in patients with and without the disease. The DLR is a useful tool to make clinical decisions with a diagnostic test, as it is inherent to the test and independent of the prevalence of the disease (26). A diagnostic test is more useful when its DLR+ value is higher, since it allows to confirm with certainty the presence of the disease, and its DLR– value is lower, since it rules out the disease (26). This concept provides clinical utility for decision-making since it has an impact on the post-test probability of the disease. In our study, the ROC curve analyses using both the actual dataset and the bootstrapping procedure yielded DLR+ >5 and DLR– <0.35 (considering both 95% CIs), which makes the value of the RT-qPCR highly clinically relevant, allowing to confirm or rule out RVA as a cause of diarrhea with high certainty (26).

Although there is information in the literature regarding the implementation of RT-qPCR Cq cutoff points for some diseases of importance in human and veterinary medicine, mainly in the former (8, 10, 27, 28), there are no studies establishing viral load cutoff points to causally associate RVA infection with disease in diarrheic calves.

A few studies carried out in domestic animals correlate LFIC or ELISA positivity in fecal samples with a Cq value range obtained by RT-qPCR (24, 25) but do not determine a cutoff point to predict causation, as some studies in humans (8, 10, 28). As highlighted before, care should be taken when interpreting Cq values as measures of the viral load (11).

As expected, the RT-qPCR was more sensitive than the ELISA to detect low viral loads since ELISA-negative calves yielded a positive result by RT-qPCR (8, 10). This indicates that a positive RT-qPCR result is not enough to demonstrate disease causality and that there is a high percentage of asymptomatic shedders that are potential sources of infection for their herd mates. As we have previously discussed (16), the lower detection rate of the ELISA can be attributed to several factors, such as its lower limit of detection and diagnostic sensitivity, and/or the presence of proteases or neutralizing antibodies in the samples that may interfere with viral detection by antigen-capture ELISA (24). This could also be related to the time of sampling, as in early stages of infection the viral load is expected to be lower, and less likely to be detected by ELISA.

An additional value of molecular testing for viral pathogens is that the amplified viral genome can then be sequenced for genotyping and/or phylogenetic analyses, which aids in the understanding of RVA molecular epidemiology (i.e., to assess transboundary dissemination or interspecies transmission), as we previously described (6).

## Conclusion

Although RT-qPCR alone cannot be used to attribute causality in cases of RVA-induced diarrhea in dairy calves, the use of a viral load cutoff point is helpful in this regard. An accurate etiologic diagnosis is crucial for the implementation of correct preventive measurements and treatment at the individual and herd level. While the cutoff point determined in this work cannot necessarily be extrapolated to other laboratories performing different nucleic acid extraction and RT-qPCR protocols, its estimation is encouraged in order to provide more comprehensive results that can be interpreted by veterinary practitioners in clinical and epidemiological contexts.

## Data availability statement

The raw data supporting the conclusions of this article will be made available by the authors, without undue reservation.

## Ethics statement

The animal study was reviewed and approved by INIA's animal Ethics Committee for the use of animals in experimentation (CEUA, protocol # 20199). Written informed consent for participation was not obtained from the owners because oral consent was obtained.

## Author contributions

RDC and FG: conceived the study, wrote the first draft, and final version of the manuscript. RDC, COS, and MLC: performed field work and conducted ELISA testing. MC and RC: performed molecular virology assays. JIA: performed statistical analyses. FG: acquired funding. All authors read, edited, and approved the final version of the manuscript.

## Funding

This work was partially funded by grants FMV_1_2014_1_104922 of the Uruguayan Agencia Nacional de Investigación e Innovación (ANII), and PL_015 N-15156 and PL_27 N-23398 of the Instituto Nacional de Investigación Agropecuaria (INIA). RDC received a master's scholarship from ANII (POS_FMV_2015_1_1005180) and a Ph.D. scholarship from INIA.

## Conflict of interest

The authors declare that the research was conducted in the absence of any commercial or financial relationships that could be construed as a potential conflict of interest. The handling editor EU declared a past co-authorship with the author FG.

## Publisher's note

All claims expressed in this article are solely those of the authors and do not necessarily represent those of their affiliated organizations, or those of the publisher, the editors and the reviewers. Any product that may be evaluated in this article, or claim that may be made by its manufacturer, is not guaranteed or endorsed by the publisher.
